# Decreased *BECN1* mRNA Expression in Human Breast Cancer is Associated With Estrogen Receptor-Negative Subtypes and Poor Prognosis

**DOI:** 10.1016/j.ebiom.2015.01.008

**Published:** 2015-01-16

**Authors:** Hao Tang, Salwa Sebti, Rossella Titone, Yunyun Zhou, Ciro Isidoro, Theodora S. Ross, Hanina Hibshoosh, Guanghua Xiao, Milton Packer, Yang Xie, Beth Levine

**Affiliations:** a Department of Clinical Sciences, University of Texas Southwestern Medical Center, Dallas, TX 75390, United States; b Center for Autophagy Research, University of Texas Southwestern Medical Center, Dallas, TX 75390, United States; c Department of Internal Medicine, University of Texas Southwestern Medical Center, Dallas, TX 75390, United States; d Laboratory of Molecular Pathology and Nanobioimaging, Department of Health Sciences, Università del Piemonte Orientale “A Avogrado”, Via Solaroli 17, 28100 Novara, Italy; e Harold C. Simmons Comprehensive Cancer Center, University of Texas Southwestern Medical Center, Dallas, TX 75390, United States; f Department of Pathology and Cell Biology, Columbia University College of Physicians & Surgeons, New York, NY 10032, United States; g Howard Hughes Medical Research Institute, University of Texas Southwestern Medical Center, Dallas, TX 75390, United States

**Keywords:** *BECN1*, *beclin 1* autophagy related gene, *BRCA1*, breast cancer 1, early onset gene, TCGA, The Cancer Genome Atlas, METABRIC, Molecular Taxonomy of Breast Cancer International Consortium, HER2, human epidermal growth factor receptor 2, *TP53*, tumor protein p53 gene, ER, estrogen receptor, GISTIC, genomic identification of significant targets in cancer, PR, progesterone receptor, PAM50, 50-gene prediction analysis of microarray, ATG5, autophagy related 5 gene, BCL-2, B-cell CLL/lymphoma 2, EGFR, epidermal growth factor receptor, OR, odds ratio, CI, confidence interval, CNV, copy-number variation, LQ, low quartile, HQ, high quartile, NA, not available, *BECN1*, *BRCA1*, Breast cancer, Autophagy

## Abstract

Both *BRCA1* and *Beclin 1* (*BECN1*) are tumor suppressor genes, which are in close proximity on the human chromosome 17q21 breast cancer tumor susceptibility locus and are often concurrently deleted. However, their importance in sporadic human breast cancer is not known. To interrogate the effects of *BECN1* and *BRCA1* in breast cancer, we studied their mRNA expression patterns in breast cancer patients from two large datasets: The Cancer Genome Atlas (TCGA) (n = 1067) and the Molecular Taxonomy of Breast Cancer International Consortium (METABRIC) (n = 1992). In both datasets, low expression of *BECN1* was more common in HER2-enriched and basal-like (mostly triple-negative) breast cancers compared to luminal A/B intrinsic tumor subtypes, and was also strongly associated with *TP53* mutations and advanced tumor grade. In contrast, there was no significant association between low *BRCA1* expression and HER2-enriched or basal-like subtypes, *TP53* mutations or tumor grade. In addition, low expression of *BECN1* (but not low *BRCA1*) was associated with poor prognosis, and *BECN1* (but not *BRCA1*) expression was an independent predictor of survival. These findings suggest that decreased mRNA expression of the autophagy gene *BECN1* may contribute to the pathogenesis and progression of HER2-enriched, basal-like, and *TP53* mutant breast cancers.

## Highlights

• The tumor suppressor genes, *BECN1* and *BRCA1*, are in close proximity to the 17q21 breast cancer tumor susceptibility locus.
• We studied mRNA expression patterns of *BECN1* and *BRCA1* in breast cancer patients in the large TCGA and METABRIC datasets.
• Decreased *BECN1* (but not *BRCA1*) expression is linked with aggressive clinico-pathological features in human breast cancer.
• Decreased *BECN1* (but not *BRCA1*) expression is linked with worse survival in human breast cancer patients.

## 1 Introduction

Estrogen-receptor (ER) negative breast cancer comprises 25–30% of all sporadic breast cancer and is characterized by advanced histological grade, aggressive clinical behavior, a high rate of metastasis to the brain and lung, and resistance to hormone deprivation therapy (Yersal and Barutca, 2014, Sorlie et al., 2001, Rakha et al., 2008a). Based on molecular profiling (Yersal and Barutca, 2014, Sotiriou and Pusztai, 2009), these cancers generally fall into two subtypes: (1) HER2-enriched tumors (those with overexpression or amplification of human epidermal growth factor receptor 2 [HER2]) and (2) basal-like tumors (which generally do not express estrogen or progesterone receptors or HER2/neu, but have high levels of basal markers and/or epidermal growth factor receptor expression and a high rate of *TP53* mutations) (Sorlie et al., 2001, Perou et al., 2000).

The pathogenesis of the basal-like subtype has not been defined, but some studies have suggested an association with dysfunction of the DNA repair *BRCA1* pathway (Turner et al., 2004, Turner et al., 2007, Mueller and Roskelley, 2003, Valentin et al., 2012). The basal-like subtype is frequent in women with *BRCA1* germline mutations (Foulkes et al., 2003) who are at markedly increased risk of breast cancer. Levels of *BRCA1* expression have also been reported to be low in women with sporadic breast cancers that have basal-like features (Turner et al., 2007, Mueller and Roskelley, 2003), and may be related to the frequent loss of heterozygosity at the breast cancer tumor susceptibility locus on chromosome 17q21 (Staff et al., 2003) and/or *BRCA1* promoter hypermethylation (Birgisdottir et al., 2006) or increased expression of negative regulatory factors (Turner et al., 2007, Garcia et al., 2011, Wu et al., 2012a).

The essential autophagy gene *beclin 1* (*BECN1*) is a haploinsufficient tumor suppressor (Liang et al., 1999, Qu et al., 2003, Yue et al., 2003) that is also located on the breast cancer tumor susceptibility chromosomal locus 17q21, ~ 150 kb centromeric to *BRCA1* (Aita et al., 1999). Monoallelic loss of *BECN1* has been observed in about 40% of human breast cancers (Aita et al., 1999, Li et al., 2010), and enforced expression of *BECN1* in breast cancer cells with allelic loss of 17q21 inhibits proliferation and tumorigenesis (Liang et al., 1999). Heterozygous deletion of *BECN1* in mice leads to an increased incidence of spontaneous carcinomas (Qu et al., 2003, Yue et al., 2003), including breast carcinoma with basal-like features (Cicchini et al., 2014).

Given the likely roles of both *BRCA1* and *BECN1* in the development of mammary malignancy and the close proximity of *BRCA1* and *BECN1* genes on chromosome 17q21, large genomic deletions of the 17q21 locus could increase the risk of sporadic breast cancer through loss of expression of both genes, or alternatively, through the loss of only one gene, with loss of the other representing a bystander effect (Laddha et al., 2014). Therefore, we sought to determine the importance of loss of *BECN1* and of *BRCA1* expression in women with ER-negative subtypes of breast cancer.

## 2 Methods

### 2.1 Genetic Profiling in Two Breast Cancer Datasets

We interrogated two large independent publicly available breast cancer datasets: The Cancer Genome Atlas Project (TCGA) in the United States (Anon., 2012) and Molecular Taxonomy of Breast Cancer International Consortium (METABRIC) in the United Kingdom and Canada (Curtis et al., 2012). The patient characteristics in each dataset are shown in Supplementary Table 1.

TCGA breast cancer mRNA gene expression, copy number alteration, and clinical data were downloaded from UCSC cancer browser at https://genome-cancer.ucsc.edu/proj/site/hgHeatmap/ (data processed in August 2014). TCGA gene expression profile was measured using the Illumina HiSeq 2000 RNA Sequencing platform. RSEM (RNA-Seq by Expectation-Maximization) normalized count was used as gene-level expression estimates in this study. TCGA copy number profile was measured using genome-wide SNP6 array. Gene-level somatic copy number alterations were estimated from TCGA FIREHOSE pipeline (https://confluence.broadinstitute.org/display/GDAC/Home) using the GISTIC2.0 (Mermel et al., 2011) method. The GISTIC2.0 summarized the copy number of each gene into − 2, − 1, 0, 1, 2, representing homozygous deletion, heterozygous deletion, diploid normal copy, low-level amplification, or high-level amplification. For analysis, the homozygous deletion and heterozygous deletion groups were combined. *TP53* mutation status was also obtained from the TCGA FIREHOSE pipeline using MutSig method (Lawrence et al., 2013). TCGA tumor grade information was manually extracted from the pathologic reports provided by the cBio portal. ER, progesterone receptor (PR), and HER2 status was defined by protein expression (measured by immunohistochemistry), as provided in the original TCGA publication; (Anon., 2012) receptor status was classified as positive, negative, or equivocal, with less than 10 samples classified as equivocal.

In the METABRIC dataset, mRNA expression was measured using the Illumina HT-12 platform. Normalized gene-level expression and copy number segment files from METABRIC were downloaded from the European Genome-phenome Archive (EGA) with accession number EGAS00000000083. The copy number profile of METABRIC data was measured using the Affymetrix SNP6 array. As for the TCGA, the copy number data of the METABRIC cohort was processed using the GISTIC2.0 (Mermel et al., 2011) algorithm to identify homozygous deletion, heterozygous deletion, diploid normal copy, low-level amplification or high-level amplification for each gene for each sample. Clinical variables were obtained from Supplementary Tables 2 and 3 of the original METABRIC publication (Curtis et al., 2012). ER, PR and HER2 status was determined by mRNA expression as positive or negative, as defined in the original METABRIC publication (Curtis et al., 2012).

Intrinsic subtyping was performed using the research-based 50-gene prediction analysis of microarray (PAM50) subtype predictor (Parker et al., 2009), which classifies tumors into the following groups: Luminal A, Luminal B, HER2-enriched, basal-like and normal-like. Samples without PAM50 data or those identified as normal-like (which often represent inadequate tumor cellularity) were excluded from the analysis. For the TCGA dataset, we used subtype calls downloaded from the database that were based on RNA-Seq measurements. For the METABRIC dataset, we used the PAM50 subtypes provided in the database; basal-like cancers were further refined into two sub-categories (IntClust categories 4 and 10) based on the clustering analysis of expression profiles as provided in the original METABRIC publication (Curtis et al., 2012).

### 2.2 Statistical Analyses

Our final analysis focused on 1067 and 1992 primary breast cancers in the TCGA and METABRIC datasets, respectively. Low versus high-expression patient groups were defined relative to the median expression level of all patients in each data set. Chi-square and Fisher's Exact tests were used to investigate the relationship between dichotomized *BECN1* or *BRCA1* expressions and PAM50 intrinsic tumor subtypes, *TP53* mutation status, advanced tumor grade, and the groups defined by ER, PR, and HER2 status. To reduce potential bias from dichotomization, the expression levels of *BECN1* and *BRCA1* were also displayed as a continuous variable and were compared across different PAM50 subtypes, *TP53* mutation status and tumor grades using a *t*-test. All cut-off values were set before analysis, and all tests were two-tailed.

Survival analysis was performed only in the METABRIC dataset because of the long median duration of follow-up (7.3 years in METABRIC and < 2 years in TCGA), using the survival R package. Patients were grouped based on the mRNA expression of *BECN1* or *BRCA1* genes, with the upper 25%, 25–75% and lower 25% representing the high, intermediate and low expression groups, respectively. Survival curves of the three groups were estimated by the Kaplan–Meier method and compared using the Cox regression model assuming an ordered trend for the three groups as previously described (Cheng et al., 2013, Shedden et al., 2008) and the log-rank test was used to compare the overall survival curves among three groups. Only deaths related to breast cancer (disease-specific deaths) were considered in the analysis. Multivariate survival analysis using the Cox regression model was performed to assess the relative contribution of *BECN1* or *BRCA1* mRNA expression, after adjusting for age, tumor grade, size, stage, molecular subtype, *TP53* mutation and perioperative therapy.

## 3 Results

### 3.1 Correlation of BECN1 and BRCA1 Deletions

*BECN1* and *BRCA1* were each deleted in approximately one-third of the breast tumors in both the TCGA and METABRIC datasets (*BECN1* deletion in 34% in TCGA and 33% in METABRIC; *BRCA1* deletion in 35% in TCGA and 27% in METABRIC) (Supplementary Table 2). The vast majority of these deletions represented heterozygous loss (350 of 354 for *BECN1* and 353 of 361 for *BRCA1* in the TCGA dataset; 493 of 643 for *BECN1* and 501 of 522 for *BRCA1* in the METABRIC dataset). As expected due to the close proximity of these two genes on chromosome 17q21, co-occurrence analysis of copy number alterations showed that the two events were highly correlated (Supplementary Table 2).

In contrast to a previous report by Laddha et al. (2014), our analyses of TCGA (n = 1033 samples) did not reveal a significant difference between the number of *BECN1* alone (n = 3) versus *BRCA1* alone deletions (n = 10) (P = 0.095). Moreover, in the METABRIC dataset (n = 1929 samples), *BECN1* alone deletions (without *BRCA1* deletions) (n = 153) were significantly more common than *BRCA1* alone deletions (without *BECN1* deletions) (n = 32) (P = 1.5E− 19). Therefore, when discordant, *BECN1* deletions were more common than *BRCA1* deletions.

**Fig. 1:**
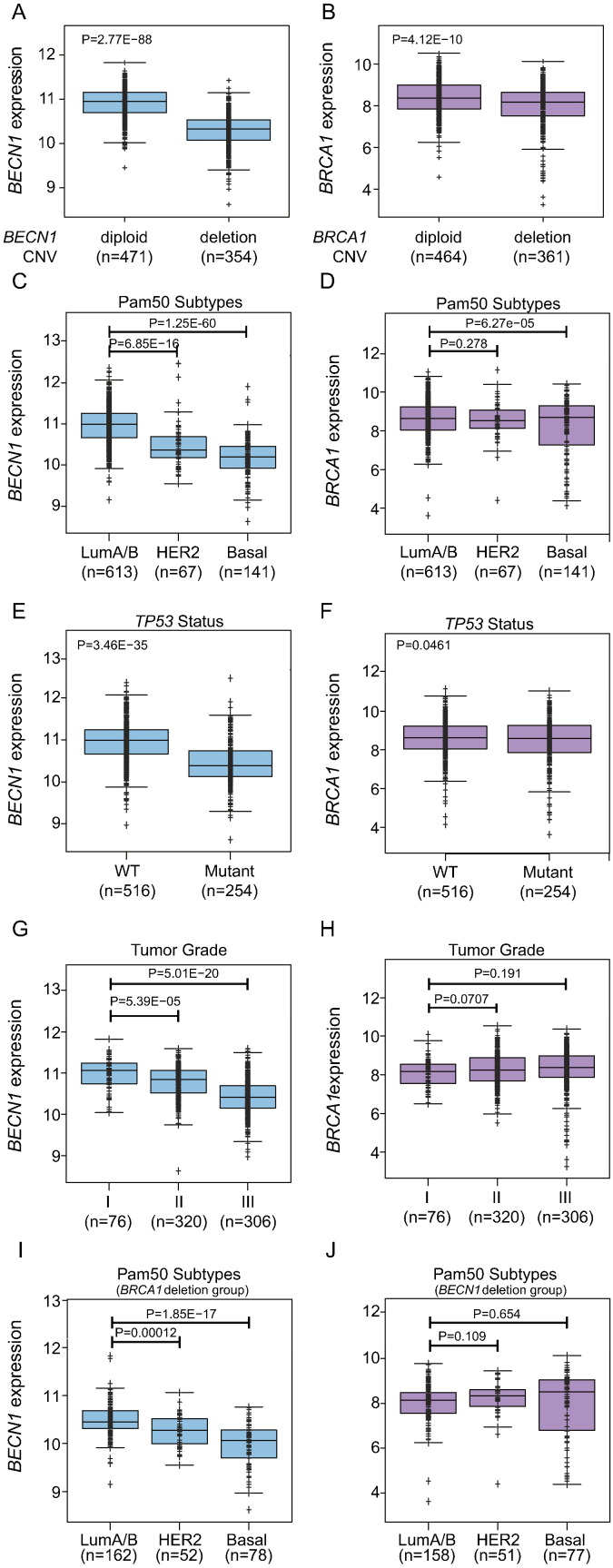
Boxplot showing the distribution of *BECN1* expression and *BRCA1* expression in TCGA, according to copy number status (panels A and B), PAM50 subtypes (panels C and D), *TP53* mutation status (panels E and F), tumor grade (panels G and H), and PAM50 subtypes in copy number loss subgroups (panels I and J). The boxes represent the median (black middle line) and the 25th–75th percentiles (lower and upper box borders). Units for gene expression represent log_2_ RSEM counts (see Methods).

**Fig. 2:**
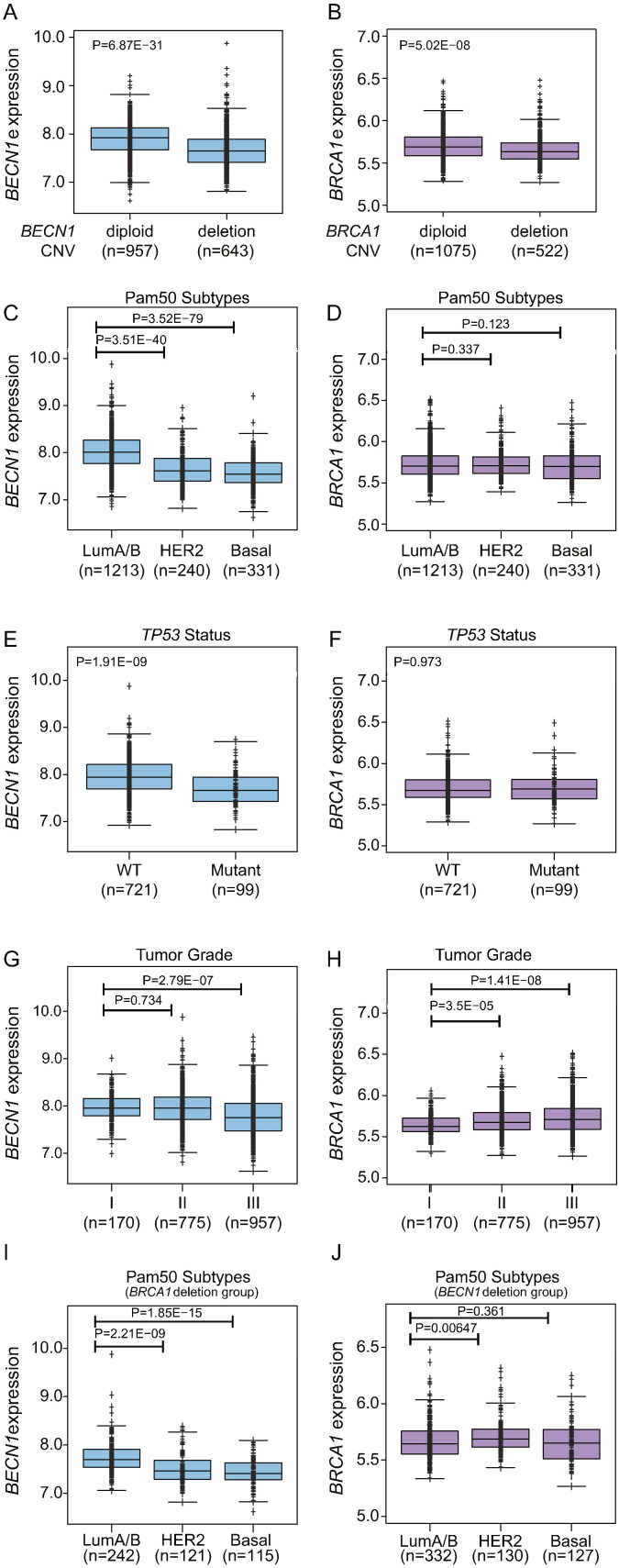
Boxplot showing the distribution of *BECN1* expression and *BRCA1* expression in METABRIC according to the copy number status (panels A and B), PAM50 subtypes (panels C and D), *TP53* mutation status (panels E and F), tumor grade (panels G and H), and PAM50 subtypes in copy number loss subgroups (panels I and J). The boxes represent the median (black middle line) and the 25th–75th percentiles (lower and upper box borders). Units for gene expression represent log_2_ intensities of Illumina array values (see Methods).

Nonetheless, since the majority of breast cancer cases with *BRCA1* or *BECN1* copy number alteration contain concurrent deletions of both *BRCA1* and *BECN1*, it is difficult to use copy number alterations as a parameter for distinguishing the effects of these two genes in breast cancer. Genes with a high correlation between their copy number and mRNA expression are more likely to be driver genes and regulate tumorigenesis, since gene expression rather than copy number better defines phenotype (Akavia et al., 2010). Notably, the relationship between copy number loss and mRNA expression was more significant for *BECN1* than for *BRCA1* in both the TCGA dataset (P = 2.77E− 88 and P = 4.12E− 10, respectively) (Fig. 1A–B) and the METABRIC dataset (P = 6.87E− 31 and P = 5.02E− 8, respectively) (Fig. 2A–B).

### 3.2 Association of low BECN1 mRNA Expression with HER2-Enriched and Basal-Like Tumor Subtypes, TP53 Mutations, and Advanced Tumor Grade

In TCGA dataset, the mRNA expression of *BECN1*, but not *BRCA1*, was associated with ER-negative intrinsic subtypes and aggressive features (Table 1). As compared with a high level of *BECN1* mRNA expression, a low level of *BECN1* mRNA expression was strongly associated with HER2-enriched breast tumors (odds ratio 8.5 [95% CI 4.4 to 17.9], P = 8.5E− 14); with basal-like breast tumors (odds ratio 35.5 [95% CI 16.4 to 91.8], P = 3.8E− 43); with the presence of *TP53* mutations (odds ratio 7.1 [95% CI 5.0 to 10.4], P = 2.6E− 32), and with tumor grade III (odds ratio 10.3 [95% CI 5.6 to 19.2], P = 2.4E− 17). In contrast, low levels of *BRCA1* mRNA expression were not significantly associated with any of these features. Low *BECN1* (but not *BRCA1*) expression was also associated with HER2-positive and triple-negative tumors identified by immunohistochemical staining (Supplementary Table 3).

In TCGA dataset, among four distinct groups with low *BECN1*/low *BRCA1*, low *BECN1*/high *BRCA1*, high *BECN1*/low *BRCA1*, and high *BECN1*/low *BRCA1* expression, only low *BECN1* expression (regardless of *BRCA1* expression) was related to the frequency of HER2 and basal-like subtypes, *TP53* mutations, and grade III tumors (Supplementary Fig. 1). Basal-like breast tumors were seen in 42.0% of patients who had low *BECN1* but high *BRCA1* expression but in only 0.7% of patients who had high *BECN1* but low *BRCA1* expression (P = 9.05E− 17 for the difference between groups) (Supplementary Fig. 1A). Similarly, as compared with the high *BECN1*/low *BRCA1* expression group, tumors with low *BECN1*/high *BRCA1* expression were more likely to be HER2-enriched (14.3% versus 0.7%, P = 4.46E− 05); have *TP53* mutations (57.5% versus 9.2%, P = 2.69E− 16); and exhibit grade III characteristics (68.5% versus 20.0%, P = 1.61E− 17) (Supplementary Fig. 1B–D).

The association between mRNA expression of *BECN1* (but not *BRCA1*) and ER-negative tumors was confirmed in the METABRIC dataset (Table 2). As compared with a high level of *BECN1* mRNA expression, a low level of *BECN1* mRNA expression was strongly associated with HER2-enriched breast tumors (odds ratio 5.5 [95% CI 4.0 to 7.7], P = 1.4E− 30); with basal-like breast tumors (odds ratio 10.0 [95% CI 7.3 to 14.1], P = 1.4E− 61); with *TP53* mutations (odds ratio 3.0 [95% CI 1.9 to 4.8], P = 8.9E− 07); and with tumor grade III (odds ratio 2.9 [95% CI 2.0 to 4.1], P = 5.8E− 10). Low levels of *BRCA1* mRNA expression were not significantly associated with any of these features, and low *BRCA1* levels were actually inversely associated with tumor grade III (odds ratio 0.4 [95% CI 0.3–0.5], P = 1.1E− 08). Low *BECN1* (but not *BRCA1*) expression was also associated with HER2-positive and triple-negative tumors identified by mRNA expression analysis (Supplementary Table 3).

Analysis of the METABRIC dataset also confirmed that low *BECN1* expression, independently of *BRCA1* expression, was associated with an increased frequency of basal-like and HER2-enriched tumors, *TP53* mutations, and tumor grade III. Basal-like breast tumors were seen in 31.0% of patients who had low *BECN1* but high *BRCA1* expression but in only 4.3% of patients who had high *BECN1* but low *BRCA1* expression (P = 3.39E − 24 for the difference between groups) (Supplementary Fig. 1E). Similarly, as compared with the high *BECN1*/low *BRCA1* expression group, tumors with low *BECN1*/high *BRCA1* expression were more likely to be HER2-enriched (21.7% versus 4.6%, P = 2.74E − 13); have *TP53* mutations (22.1% versus 6.7%, P = 4.32E − 05); and exhibit grade III characteristics (65.7% versus 29.8%, P = 1.19E − 24) (Supplementary Fig. 1F–H).

These findings were confirmed when gene expression was compared across different tumor subtypes without dichotomization. In TCGA, *BECN1* (but not *BRCA1*) expression was significantly lower in basal-like (P = 1.25E− 60) and HER2-enriched tumors (P = 6.85E− 16), tumors with *TP53* mutations (P = 3.46E− 35), and tumors with an advanced histological grade (P = 5.39E− 05 for grade II, P = 5.01E− 20 for grade III) (Fig. 1C–H). Similarly, in the METABRIC cohort, *BECN1* (but not *BRCA1*) expression was also significantly lower in basal-like (P = 3.52E− 79) and HER2-enriched tumors (P = 3.51E− 40), tumors with *TP53* mutations (P = 1.91E− 09), and tumors with an advanced histological grade (P = 2.79E− 07 for grade III) (Fig. 2C–H). In the TCGA (but not in METABRIC) dataset, despite higher median values for *BRCA1* expression in basal-like tumors, a small proportion had very low levels of *BRCA1* expression (Fig. 1D).

Similar results confirmed the association of low *BECN1* expression with ER-negative tumor subtypes when the analyses were confined to tumors with *BRCA1* deletions. In TCGA dataset (Supplementary Table 4), low *BECN1* expression was associated with basal-like breast tumors (odds ratio 8.3 [95% CI 4.2 to 17.3], P = 3.8E− 12), HER2-enriched breast tumors (odds ratio 3.5 [95% CI 1.7 to 7.1], P = 1.7E− 04), and tumors with *TP53* mutation (odds ratio 3 · 1 [95% CI 1.8 to 5.3], P = 1.0E− 05). In the METABRIC dataset (Supplementary Table 4), low *BECN1* expression was associated with basal-like breast tumors (odds ratio 5.6 [95% CI 3.4 to 9.6], P = 5.5E− 13), HER2-enriched breast tumors (odds ratio 4.1 [95% CI 2.5 to 6.7], P = 8.8E− 10), tumors with *TP53* mutation (odds ratio 2.3 [95% CI 1.1 to 4.9], P = 0.017), and grade III tumors (odds ratio 3.4 [95% CI 1.5–8.5], P = 0.002). In both the TCGA and METABRIC datasets, in the *BECN1* deletion subgroup, low *BRCA1* expression was not associated with ER-negative tumor subtypes, *TP53* mutations or advanced tumor grade; in fact, in METABRIC, low *BRCA1* expression was inversely associated with HER2-enriched (odds ratio 0.5 [95% CI 0.3–0.8], P = 0.0018) and grade III tumors (odds ratio 0.3 [95% CI 0.2–0.6], P5.5E− 04) (Supplementary Table 4). In both TCGA and METABRIC, when gene expression was compared across different tumor types without dichotomization, *BECN1* expression in the *BRCA1* deletion group was significantly lower in HER2-enriched tumors (P = 0.00012 and P = 2.21E– 09, respectively) and basal-like tumors (P = 1.85E– 17 and P = 1.85E– 15, respectively) (Fig. 1I, Fig. 2I). In contrast, there was no association between low *BRCA1* expression and these ER-negative PAM50 subtypes in the *BECN1* deletion group (Fig. 1J, Fig. 2J).

In a subgroup analysis of patients who were diploid for *BECN1* and *BRCA1*, in both the TCGA and METABRIC datasets, we also found that low *BECN1* mRNA expression but not low *BRCA1* mRNA expression was associated with HER2-enriched and basal-like tumor subtypes, *TP53* mutations, and grade III tumors (Supplementary Table 5). Moreover, for both TCGA and METABRIC datasets, low *BECN1* expression was associated with these same features in patients with high *BRCA1* expression (Supplementary Table 6) or low *BRCA1* expression (Supplementary Table 7). In contrast, low *BRCA1* expression was not positively associated with any of these features in high *BECN1* or low *BECN1* expression subgroups (Supplementary Tables 6 and 7). Low *BRCA1* expression was actually inversely associated with several of these features; however, the only associations which were significant in both the TCGA and METABRIC datasets were between low *BRCA1* expression and a reduced odds of grade III tumors. This was observed in both patients with high *BECN1* expression (Supplementary Table 6) or low *BECN1* expression (Supplementary Table 7).

### 3.3 Association Between Low BECN1 mRNA Expression and Worse Patient Survival

**Fig. 3:**
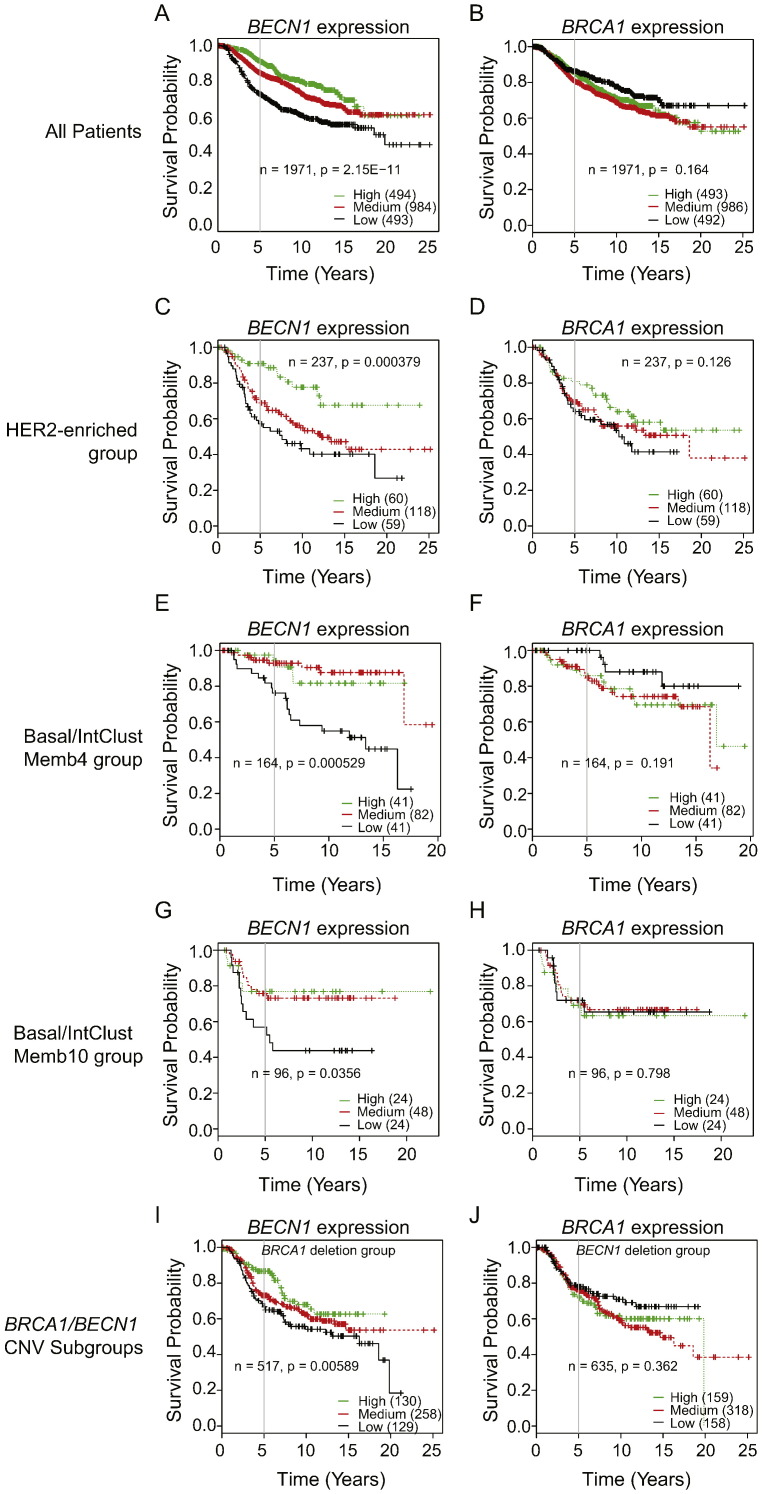
*BECN1* expression but not *BRCA1* expression is associated with disease-specific survival. Panels A and B: Kaplan–Meier curves for all patients for *BECN1* expression (panel A) or *BRCA1* (panel B) expression. Panels C through J: Kaplan–Meier curves within HER2-enriched group (panels C and D), Basal/IntClust Memb4 (panels E and F), Basal/IntClust Memb10 (panels G and H), and in copy number loss subgroups (panels I and J). Green, red and black lines indicate high (1st quartile), medium (2nd and 3rd quartiles), and low (4th quartile) expression level groups, respectively. + denotes censored observations. P values were obtained by the Cox regression model assuming an ordered trend for the three expression groups.

Patients whose tumors had the lowest levels of *BECN1* expression had the worst prognosis (P = 2.15E− 11) (Fig. 3A). In contrast, the level of *BRCA1* expression was not associated with survival (P = 0.164). Similar results were observed when the analyses were restricted to ER-negative intrinsic subtypes. Overall, the level of *BECN1* expression was directly associated with length of survival in patients with HER2-enriched tumors (P = 3.79E− 04) (Fig. 3C), basal-like with IntClust 4 (P = 5.29E− 04) (Fig. 3E) and basal-like with IntClust 10 (P = 0.036) (Fig. 3G). In contrast, *BRCA1* expression was not associated with survival in HER2-enriched tumors or in the two basal-like subgroups (Fig. 3D, F, and H).

By multivariate analysis, low *BECN1* expression was significantly associated with shortened survival, even after adjustment for *BRCA1* expression, age, tumor grade, tumor size, stage, intrinsic subtypes, *TP53* mutation and treatment (hazard ratio 0.6 [0.4–0.9], P = 0.02) (Table 3). Furthermore, in the *BRCA1* deletion subgroup, patients with low *BECN1* expression had a significantly worse survival than those with high *BECN1* expression (P = 0.00589) (Fig. 3I), whereas in the *BECN1* deletion subgroup, there was no significant relationship between high and low levels of *BRCA1* expression and survival (Fig. 3J).

## 4 Discussion

As expected due to their close proximity on chromosome 17q21, *BECN1* and *BRCA1* are often concordantly deleted or amplified in breast cancers. However, our findings indicate that decreased *BECN1* (but not decreased *BRCA1*) expression characterizes breast cancers that have aggressive molecular and clinical characteristics. When compared with tumors with high levels of expression, tumors with low *BECN1* expression were more likely to have a higher histological grade, *TP53* mutations, HER2-enriched or basal-like intrinsic subtypes, triple-negative status, and worse survival. In contrast, the levels of *BRCA1* expression did not distinguish tumors with these aggressive characteristics or unfavorable prognosis. Furthermore, in tumors with deletion of *BRCA1*, levels of *BECN1* expression provided important additional discriminatory information; however, in tumors with deletion of *BECN1*, levels of *BRCA1* expression did not distinguish the molecular and clinical features of tumors. Importantly, these relationships were observed across two independent regional databases with different expression analysis platforms (RNA-seq and microarray), suggesting that our results cannot be explained by population differences or idiosyncrasies in the characterization of tumors.

Our findings are consistent with earlier studies of *BECN1* in small cohorts of patients with breast cancer. Levels of *BECN1* mRNA expression have been reported to be reduced in breast cancer (Li et al., 2010, Wu et al., 2012b) and have been associated with poor differentiation, and increased tumor size, proliferation and risk of metastasis (Wu et al., 2012b, Yao et al., 2011). In small datasets, low *BECN1* mRNA expression was associated with triple-negative breast cancer (Cicchini et al., 2014) and with worse prognosis regardless of ER status (Perou et al., 2000, Dong et al., 2013). In addition, *BECN1* DNA copy number loss has been reported to be associated with HER2 amplification and *TP53* mutations (Negri et al., 2010).

One previous analysis of TCGA dataset by Laddha et al. (2014) reported deletions of *BRCA1* alone but not *BECN1* alone in human breast cancer. That study, however, used an ad hoc heuristic approach for identifying deletions; our analyses of copy number variations based on the more rigorous GISTIC method could not confirm this earlier report. In fact, in METABRIC, *BECN1* alone deletions were more common than *BRCA1* deletions, indicating a further lack of confirmation of the findings of Laddha et al. In addition, Laddha et al. reported that there were no changes in the mean level of *BECN1* mRNA expression in breast tumor samples versus normal tissue. However, the validity of this comparison is difficult to assess, since epithelial cells (which have very high levels of *BECN1* expression) comprise the majority of cells in tumor samples but only a small proportion of cells in normal breast tissue. Most importantly, Laddha et al. considered human breast cancer to be a homogenous disease and did not analyze the relationship between *BECN1* mRNA expression and specific clinical and pathological features of breast cancer. Our analyses of two large datasets, TCGA and METABRIC, revealed a marked association between low *BECN1* expression and ER-negative breast cancers subtypes with aggressive clinical features.

Our finding that low *BRCA1* expression was not associated with basal-like subtype or worse survival is consistent with the lack of evidence that somatic loss of *BRCA1* contributes meaningfully to sporadic breast cancer. Only homozygous, not heterozygous, *Brca1* knockout mice develop breast cancers (Evers and Jonkers, 2006), whereas breast (and other) cancers develop in *Becn1* heterozygous knockout mice (Qu et al., 2003, Yue et al., 2003, Cicchini et al., 2014). Moreover, loss of *BRCA1* heterozygosity in humans with germline *BRCA1* mutations is necessary for the development of BRCA1 mutant-associated breast cancers (Futreal et al., 1994). This is likely because haploinsufficient *BRCA1* expression is sufficient for full DNA repair (Latimer et al., 2005). Thus, given the rare frequency of somatic *BRCA1* mutations (despite the high prevalence of *BRCA1* heterozygous loss) (Futreal et al., 1994), a role for *BRCA1* deficiency in sporadic breast cancer is not established.

Nonetheless, previous studies have shown similarities between the clinical and molecular features of sporadic basal-like tumors and familial *BRCA1*-mutated tumors, resulting in the model that basal-like tumors may be associated with *BRCA1* dysfunction (Turner et al., 2004, Turner et al., 2007, Valentin et al., 2012, Turner and Reis-Filho, 2006). Low *BRCA1* expression and/or *BRCA1* promoter methylation has been associated with basal-like sporadic breast cancers in some reports (Turner et al., 2007, Joosse et al., 2011, Lee et al., 2010, Rakha et al., 2008b), but not others (Matros et al., 2005, Richardson et al., 2006). Regardless of their findings, these studies generally analyzed small numbers of patients; did not identify tumor subtypes by molecular profiling; and identified low *BRCA1* samples using immunohistochemical staining for protein expression or quantitative PCR for mRNA expression, which are both subject to difficulties in standardization and reproducibility. Our study is the first to apply current state-of-the-art methods for *BRCA1* mRNA quantification to a large number of samples characterized by intrinsic molecular subtypes. Our inability to find a relation between *BRCA1* expression and basal-like breast cancers supports the concept that the phenotypic similarities of sporadic basal-like breast tumors and hereditary *BRCA1* mutated tumors may be explained by factors other than *BRCA1* dysfunction (Matros et al., 2005). Alternatively, our data (Fig. 1D) suggests that low *BRCA1* expression may characterize only a small subgroup of basal-like tumors, whose specific features are yet to be defined. Another possible explanation is that other factors, besides somatic mutations or decreased mRNA expression (either as a result of copy number variation or epigenetic regulation), act to impair *BRCA1* function in sporadic breast cancer. Thus, although our results consistently show a lack of relationship between decreased *BRCA1* expression and basal-like breast cancer, they cannot definitively exclude a role for *BRCA1* dysfunction in sporadic basal-like breast cancer.

We propose that the decreased expression of *BECN1* (another tumor suppressor gene located near *BRCA1*) in sporadic basal-like breast tumors may partly explain the phenotypic overlap of this disease with hereditary *BRCA1* breast cancer. Patients with germline mutations in *BRCA1* usually have somatic deletion of wild-type chromosome 17q21 in their breast tumors; (Turner et al., 2004, Palacios et al., 2008) thus, the co-deletion of *BECN1* in such cases may contribute to the development of basal-like features. Independently of whether the co-deletion of *BECN1* plays a role in hereditary *BRCA1* breast cancer, decreased *BECN1* expression — which results in reduced levels of autophagy (Qu et al., 2003) — may exert effects on the DNA damage repair pathway in sporadic breast cancer similar to those produced by a *BRCA1* mutation and loss of heterozygosity in hereditary breast cancer. In support of this theory, knockdown of another essential autophagy gene, *ATG5*, suppresses the expression of RAD51, a key protein that functions in homologous recombination and repair of DNA double-stranded breaks (Mo et al., 2014).

Taken together, our findings suggest that decreased *BECN1* expression may contribute to the pathogenesis and/or progression of certain breast cancers, especially the ER-negative subtypes. A deficiency of *BECN1* leads to defects in autophagy (Qu et al., 2003), a lysosomal degradation “housekeeping” pathway that prevents chromosomal instability and DNA damage and inhibits cellular proliferation; (Levine and Kroemer, 2008) alternatively, loss of other functions of *BECN1* (e.g., receptor endocytosis) (Funderburk et al., 2010) may play a role in carcinogenesis. Future clinical trials should evaluate whether the level of *BECN1* expression predicts the response to specific chemotherapeutic regimens or whether strategies that increase *BECN1* function might be therapeutic in patients with low *BECN1* expression. Of note, the autophagy activity of Beclin 1 is inhibited by interaction with BCL-2 family members (Pattingre et al., 2005, Maiuri et al., 2007), by oncogenic kinase AKT and EGFR-mediated Beclin 1 post-translational modifications (Wang et al., 2012, Wei et al., 2013), and by interactions with HER2 (Han et al., 2013). Thus, currently available Beclin 1/BCL-2 binding inhibitors, AKT inhibitors, EGFR inhibitors and HER2 inhibitors may act to increase Beclin 1 function in tumors with low *BECN1* expression, and thereby, improve clinical outcomes.

## Role of the Funding Source

This work was supported by NIH grants R01CA152301 (Y.X. and G.X.), R01CA172211 (Y.X.), 5P50CA70907 (Y.X.) and RO1CA109618 (B.L.); and the Cancer Prevention Research Institute of Texas awards RP101251 (Y.X.) and RP120718 (B.L.). These funders had no role in the study design, data collection, data analysis, interpretation or writing of the report.

## Author Contributions

HT, SS, MP, YX, and BL contributed to the study design, data analysis, data interpretation and writing of the report. RT contributed to the study design and data analysis. YZ and GX contributed to data analysis. CI and HH contributed to the study design and data interpretation. TR contributed to data interpretation and writing of the report.

## Declaration of Interests

Beth Levine has received consulting fees from Novus Biologicals.

## Figures and Tables

**Table 1:** *BECN1* and *BRCA1* expression association with clinical features (TCGA cohort).

	*BECN1* expression				*BRCA1* expression			
mRNA expression		Odds ratio (95% confidence interval)	P value	mRNA expression		Odds ratio (95% confidence interval)	P value
High	Low	High	Low
*PAM50 subtypes*								
Luminal A/B	399	214	Reference		348	265	Reference	
HER2-enriched	12	55	8.5 [4.4, 17.9]	8.5E− 14	37	30	1.1 [0.6, 1.8]	0.90
Basal-like	7	134	35.5 [16.4, 91.8]	3.8E− 43	82	59	0.9 [0.6, 1.4]	0.78
*TP53 mutation*								
Wild type	337	179	Reference	2.6E− 32	300	216	Reference	
Mutant	53	201	7.1 [5.0, 10.4]		138	116	1.1 [0.8, 1.5]	0.49
*Tumor grade*								
I	55	21	Reference	0.003	25	51	Reference	
II	171	149	2.3 [1.3, 4.2]	2.4E− 17	133	187	0.7 [0.4, 1.2]	0.19
III	62	244	10.3 [5.6, 19.2]		145	161	0.5 [0.3, 0.9]	0.03

**Table 2:** *BECN1* and *BRCA1* expression association with clinical features (METABRIC cohort).

	*BECN1* expression				*BRCA1* expression			
mRNA expression		Odds ratio (95% confidence interval)	P value	mRNA expression		Odds ratio (95% confidence interval)	P value
High	Low	High	Low
*PAM50 subtypes*								
Luminal A/B	804	409	Reference		656	557	Reference	
HER2-enriched	63	177	5.5 [4.0, 7.7]	1.4E− 30	136	104	0.9 [0.7, 1.2]	0.48
Basal-like	54	277	10.0 [7.3, 14.1]	1.4E− 61	168	163	1.1 [0.9, 1.5]	0.29
*TP53 mutation*								
Wild type	423	298	Reference		345	376	Reference	0.52
Mutant	32	67	3.0 [1.9, 4.8]	8.9E− 07	51	48	0.9 [0.6, 1.3]	
*Tumor grade*								
I	111	59	Reference		53	117	Reference	
II	462	313	1.3 [0.9, 1.8]	0.194	371	404	0.5 [0.3, 0.7]	8.3E− 05
III	379	578	2.9 [2.0, 4.1]	5.8E− 10	526	431	0.4 [0.3, 0.5]	1.1E− 08

**Table 3:** Multivariate survival analysis.⁎

	Hazards ratio (95% confidence interval)	P value
*BRCA1 expression*	1.0 (0.4, 2.2)	0.95
*BECN1 expression*	0.6 (0.4, 0.9)	0.02
*Age at diagnosis*	1.02 (1.00, 1.03)	0.03
*Tumor grade*		
I	Reference	
II	1.0 (0.5, 2.2)	0.94
III	1.4 (0.7, 3.1)	0.37
*Tumor size*		
< = 20 (T1)	Reference	
20–50 (T2)	1.6 (1.1, 2.2)	0.01
> 50 (T3)	1.5 (0.7, 3.3)	0.27
*Tumor stage*		
Stage 0	Reference	
Stage 1	0.8 (0.5, 1.2)	0.31
Stage 2	0.5 (0.3, 0.8)	2.8E− 03
Stage 3	1.8 (1.0, 3.2)	0.06
Stage 4	1.7 (0.4, 7.4)	0.46
*PAM50 subtype*		
Luminal A/B	Reference	
HER2-enriched	0.9 (0.5, 1.6)	0.68
Basal-like	0.6 (0.3, 1.1)	0.07
*TP53 mutation status*		
Wild type	Reference	
Mutant	2.1 (1.5, 3.0)	7.4E− 05
*Treatment*		
Radiation therapy	Reference	
Hormonal therapy	1.0 (0.5, 2.0)	0.94
Hormonal/radiation therapy	1.5 (0.8, 2.9)	0.20
Chemotherapy	1.5 (0.8, 2.8)	0.17
Chemotherapy/radiation therapy	6.8 (2.6, 17.8)	8.1E− 05
Chemotherapy/hormonal therapy	5.4 (2.3, 12.6)	8.7E− 05
Chemotherapy/hormonal/radiation therapy	2.2 (0.5, 10.4)	0.32
Radiation therapy	2.3 (1.1, 4.9)	0.04

⁎ Multivariate Cox regression model was performed to assess the relative contribution of *BECN1* or *BRCA1* mRNA expression in predicting prognosis, after adjusting for other clinical factors listed in the table. To reduce potential bias from dichotomization, continuous gene expression values were used.
