# Supplementary material for: Decreased BECN1 mRNA Expression in Human Breast Cancer is Associated With Estrogen Receptor-Negative Subtypes and Poor Prognosis
Source: eBioMedicine. 2015 Jan 16;2(3):255–63. doi: 10.1016/j.ebiom.2015.01.008 (PMC4376376; doi:10.1016/j.ebiom.2015.01.008)
Supplement: Supplementary file 1 — Supplementary material. [file mmc1.pdf]

SUPPLEMENTAL DATA

Supplementary Figure Legends 1-2
Supplementary Figures 1-2
Supplementary Tables 1-7

Supplementary Figure Legends

Supplementary Figure 1. Frequency of basal subtype (Panels A, E), HER2 subtype (Panels B, F), *TP53* mutations (Panels C, G), and grade III tumors (Panels D, H) in the TCGA (Panels A-D) and METABRIC (Panels E-H) datasets among patients with low *BECN1*/low *BRCA1,* low *BECN1*/high *BRCA1*, high *BECN1*/low *BRCA1* and high *BECN1*/high *BRCA1* expression. *BECN1* and *BRCA1* expression values were analyzed as binary variables split by the median value. Altogether, the numbers of samples in the low *BRCA1*/low *BECN1* or high *BRCA1*/high *BECN1* group are 325 in TCGA and 563 in METABRIC; the numbers of samples in the high *BRCA1*/low *BECN1* or low *BRCA1*/high *BECN1* group are 206 in TCGA and 433 in METABRIC.

Supplementary Figure 2. Boxplot showing the distribution of *BECN1* expression and *BRCA1* expression in patients with Luminal A/B, HER2+ER- and triple negative status in the TCGA dataset (Panels A and B) and METABRIC dataset (Panels C and D). The boxes represent the median (black middle line) and the 25th-75th percentiles (lower and upper box borders).). Vertical lines represent the upper (25th percentile – IQR) and lower (75th percentile – IQR) extremes. Units for gene expression represent $\log_2$ RSEM counts for TCGA data and $\log_2$ Illumina array values for METABRIC data.

Supplementary Figure 1
Tang et al.

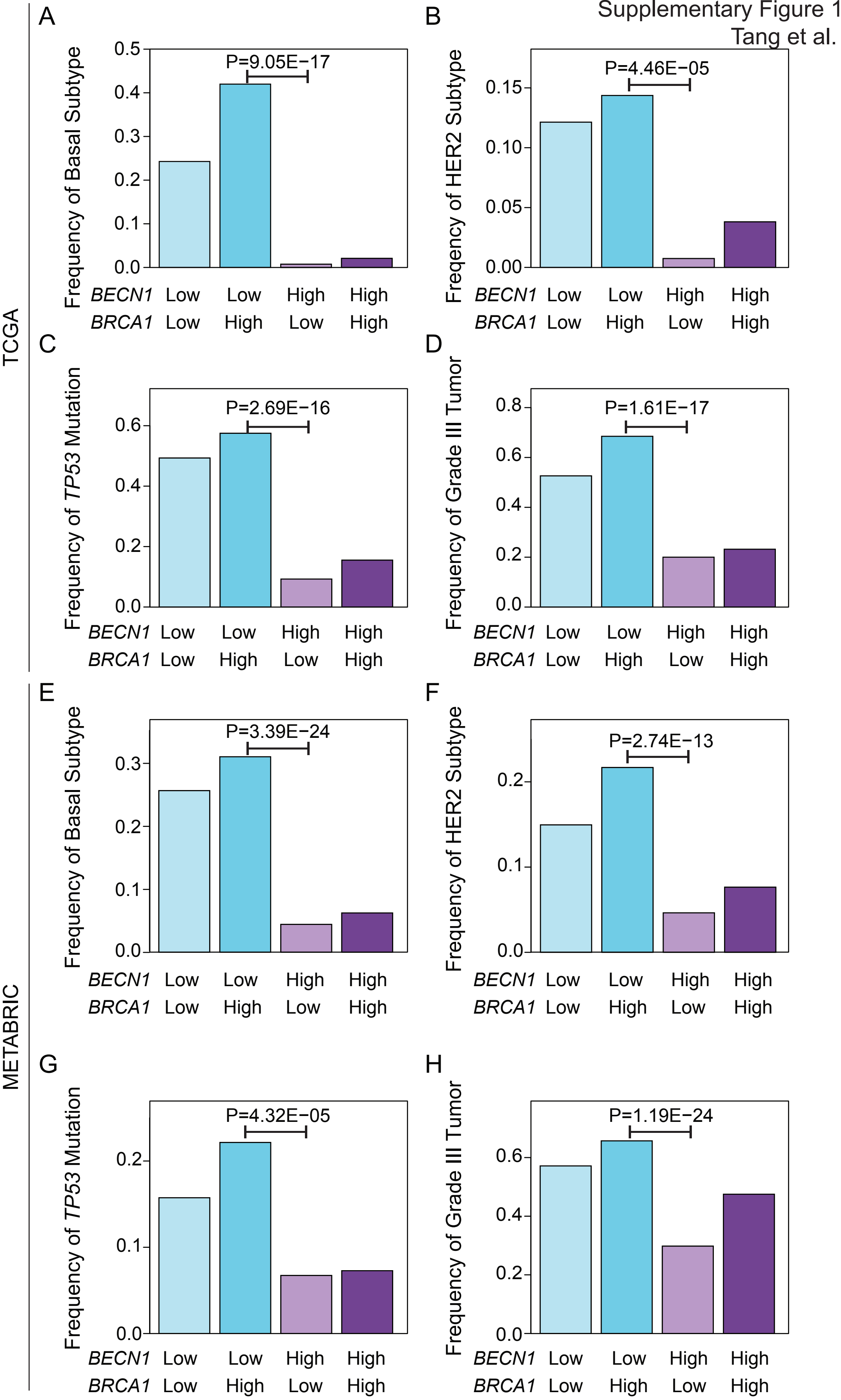

Supplementary Figure 2
Tang et al.

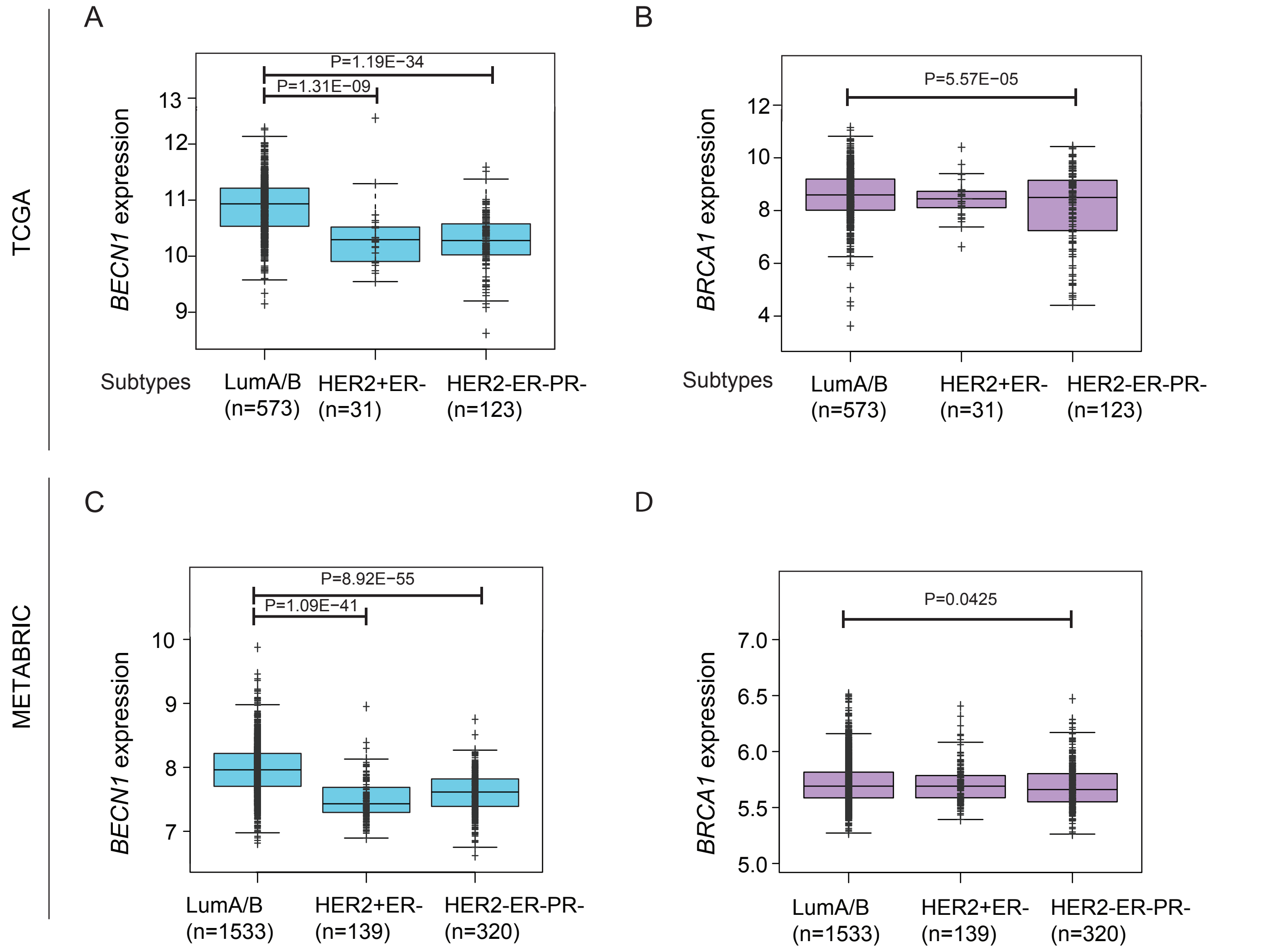

Supplementary Table 1. Patient characteristics in TCGA and METABRIC cohorts.

	TCGA		METABRIC	
**Clinical Characteristics**	**Number**	**Percent**	**Number**	**Percent**
**Age at Diagnosis (years)**(Median [LQ-HQ])	58.0 [49.0-67.0]		61.8 [51.4-70.6]	
**Follow Up (years)**(Median [LQ-HQ])	1.6 [0.8-3.5]		7.2 [4.1-11.9]	
**Survival**				
Alive at time of last follow-up or death due to unrelated cause	910	85.3	1466	73.6
Disease-specific deaths	110	10.3	506	25.4
NA	47	4.4	20	1.0
**Tumor Size**				
<=20mm (T1)	256	24.83	862	43.3
20-50mm (T2)	581	56.35	1008	50.6
>50mm (T3)	155	15.03	102	5.1
NA	39	3.78	20	1.0
**Number of Lymph Nodes Involved**				
0 (N0)	481	45.1	1042	52.3
1-3 (N1)	338	31.7	625	31.4
4-9 (N2)	113	10.6	227	11.4
>9 (N3)	72	6.8	91	4.6
NA	63	5.9	7	0.4
**Tumor Grade (Nottingham)**				
I	77	7.2	170	8.5
II	320	30.0	775	38.9
III	306	28.7	957	48.0
NA	364	34.1	90	4.5
***BRCA1* CNV**				
Gain/Amplification	207	19.4	332	16.7
Diploid	465	43.6	1075	54.0
Heterozygous/Homozygous Deletion	361	33.8	522	26.2
NA	34	3.2	63	3.2
***BECN1* CNV**				
Gain/Amplification	207	19.4	329	16.5
Diploid	472	44.2	957	48.0
Heterozygous/Homozygous Deletion	354	33.2	643	32.3
NA	34	3.2	63	3.2
**ER Status (IHC)**				
Negative	179	16.8	474	23.8
Positive	601	56.3	1518	76.2
NA	287	26.9		
***TP53* Mutation Status**				
Wild type	516	48.4	721	36.2
Mutant	254	23.8	99	5.0
NA	297	27.8	1172	58.8
**PAM50 Subtype**				
Normal	23	2.2	202	10.1
Luminal B	192	18.0	492	24.7
Luminal A	421	39.5	721	36.2
HER2-enriched	67	6.3	240	12.0
Basal-like	141	13.2	331	16.6
Not classified	223	20.9	6	0.3
**Treatment**				
NONE	445	41.7	309	15.5
Radiation therapy	22	2.1	232	11.7
Hormonal therapy	62	5.8	420	21.1
Hormonal/radiation therapy	65	6.1	609	30.6
Chemotherapy	69	6.5	53	2.7
Chemotherapy/radiation therapy	106	9.9	173	8.7
Chemotherapy/hormonal therapy	78	7.3	31	1.6
Chemotherapy/hormonal/radiation therapy	173	16.2	165	8.3
NA	47	4.4		

Supplementary Table 2. CNV value relations of *BECN1* and *BRCA1.*

TCGA Cohort

		***BRCA1* CNV**				
		Amplification	Gain	Diploid	Heterozygous Loss	Homozygous Deletion
***BECN1* CNV**	Amplification	13	0	0	0	0
	Gain	1	187	0	5	1
	Diploid	2	1	465	3	1
	Heterozygous Loss	0	3	0	345	2
	Homozygous Deletion	0	0	0	0	4

METABRIC Cohort

		***BRCA1* CNV**				
		Amplification	Gain	Diploid	Heterozygous Loss	Homozygous Deletion
***BECN1* CNV**	Amplification	8	5	6	6	0
	Gain	1	267	26	10	0
	Diploid	3	12	926	15	1
	Heterozygous Loss	3	14	36	436	4
	Homozygous Deletion	1	18	81	34	16

Supplementary Table 3. *BECN1* and *BRCA1* expression association with ER, HER2, and PR status.

TCGA Cohort

	BECN1				***BRCA1***			
	**mRNA Expression**		**Odds Ratio (95% confidence intervals)**	**P Value**	**mRNA Expression**		**Odds Ratio (95% confidence intervals)**	**P Value**
	High	Low			High	Low		
**Subtypes**								
Luminal A/B	338	235	Reference		319	254	Reference	
HER2+ER-	2	29	20.8 [5.2, 181.1]	2.6E-09	16	15	1.2 [0.5, 2.6]	0.71
HER2-ER-PR-	12	111	13.3 [7.1, 27.1]	2.0E-25	64	59	1.2 [0.8, 1.7]	0.49

METABRIC Cohort

	BECN1				***BRCA1***			
	**mRNA Expression**		**Odds Ratio (95% confidence intervals)**	**P Value**	**mRNA Expression**		**Odds Ratio (95% confidence intervals)**	**P Value**
	High	Low			High	Low		
**Subtypes**								
Luminal A/B	920	613	Reference		777	765	Reference	
HER2+ER-	18	121	10.1 [6.0, 17.8]	3.2E-28	73	66	0.9 [0.6, 1.3]	0.72
HER2-ER-PR-	58	262	6.8 [5.0, 9.3]	1.6E-44	146	174	1.2 [1.0, 1.6]	0.11

Supplementary Table 4. *BECN1* and *BRCA1* expression association with subtypes among patients with copy number loss.

TCGA cohort

	***BECN1* expression in *BRCA1* deletion group**				***BRCA1* expression in *BECN1* deletion group**			
	**mRNA Expression**		**Odds Ratio (95% confidence intervals)**	**P Value**	**mRNA Expression**		**Odds Ratio (95% confidence intervals)**	**P Value**
	High	Low			High	Low		
**PAM50 Subtypes**								
Luminal A/B	108	54	Reference		77	81	Reference	
HER2-enriched	19	33	3.5 [1.7, 7.1]	1.7E-4	33	18	0.5 [0.3, 1.0]	0.054
Basal-like	15	63	8.3 [4.2, 17.3]	3.8E-12	47	30	0.6 [0.3, 1.1]	0.095
***TP53* Mutation**								
Wild type	78	41	Reference		57	59	Reference	
Mutant	59	96	3.1 [1.8, 5.3]	1.0E-05	92	60	0.6 [0.4, 1.1]	0.082
**Tumor Grade**								
I	8	3	Reference		3	7	Reference	
II	63	43	1.8 [0.4, 11.2]	0.520	43	61	0.6 [0.1, 2.9]	0.740
III	82	107	3.5 [0.8, 20.9]	0.069	114	77	0.3 [0.05, 1.3]	0.098

METABRIC cohort

	***BECN1* expression in *BRCA1* deletion group**				***BRCA1* expression in *BECN1* deletion group**			
	**mRNA expression**		**Odds Ratio (95% confidence intervals)**	**P Value**	**mRNA expression**		**Odds Ratio (95% confidence intervals)**	**P Value**
	High	Low			High	Low		
**PAM50 Subtypes**								
Luminal A/B	166	76	Reference		165	167	Reference	
HER2-enriched	42	79	4.1 [2.5, 6.7]	8.8E-10	86	44	0.5 [0.3, 0.8]	0.0018
Basal-like	32	83	5.6 [3.4, 9.6]	5.5E-13	64	63	1.0 [0.6, 1.5]	0.920
***TP53* Mutation**								
Wild type	108	63	Reference		98	112	Reference	
Mutant	19	26	2.3 [1.1, 4.9]	0.017	26	23	0.8 [0.4, 1.5]	0.430
**Tumor Grade**								
I	26	9	Reference		15	37	Reference	
II	83	69	2.4 [1.0, 6.2]	0.037	95	109	0.5 [0.2, 0.9]	0.028
III	148	175	3.4 [1.5, 8.5]	0.002	204	167	0.3 [0.2, 0.6]	5.5E-04

Supplementary Table 5. *BECN1* and *BRCA1* expression association with subtypes among patients with diploid copy number.

TCGA cohort

	BECN1 expression in *BRCA1* diploid group				*BRCA1* expression in *BECN1* diploid group			
	mRNA Expression		Odds Ratio (95% confidence intervals)	P Value	mRNA Expression		Odds Ratio (95% confidence intervals)	P Value
	High	Low			High	Low		
**PAM50 Subtypes**								
Luminal A/B	177	110	Reference		176	117	Reference	
HER2-enriched	2	6	4.8 [0.8, 49.5]	0.061	7	1	0.2 [0.005, 1.7]	0.16
Basal-like	0	43	Inf* [17.4, Inf]	1.1E-16	29	14	0.7 [0.3, 1.5]	0.41
***TP53* Mutation**								
Wild type	152	111	Reference		158	109	Reference	
Mutant	14	41	4.0 [2.0, 8.3]	1.3E-05	34	22	0.9 [0.5, 1.8]	0.88
**Tumor Grade**								
I	49	16	Reference		26	40	Reference	
II	117	95	2.5 [1.3, 5.0]	0.0038	109	107	0.6 [0.3, 1.2]	0.12
III	34	80	7.1 [3.4, 15.4]	4.2E-09	68	47	0.4 [0.2, 0.9]	0.013

*Inf, infinity (positive)

METABRIC cohort

	BECN1 expression in *BRCA1* diploid group				*BRCA1* expression in *BECN1* diploid group			
	mRNA Expression		Odds Ratio (95% confidence intervals)	P Value	mRNA Expression		Odds Ratio (95% confidence intervals)	P Value
	High	Low			High	Low		
**PAM50 Subtypes**								
Luminal A/B	441	238	Reference		324	274	Reference	
HER2-enriched	22	60	5.0 [3.0, 8.9]	4.7E-11	44	29	0.8 [0.5, 1.3]	0.383
Basal-like	29	140	8.9 [5.8, 14.3]	4.2E-30	90	64	0.8 [0.6, 1.2]	0.364
***TP53* Mutation**								
Wild type	221	162	Reference		162	191	Reference	
Mutant	9	23	3.45[1.5, 8.8]	0.0015	19	10	0.4 [0.2, 1.0]	0.05
**Tumor Grade**								
I	72	48	Reference		35	68	Reference	
II	264	207	1.2 [0.8, 1.8]	0.47	193	225	0.6 [0.4, 1.0]	0.027
III	171	248	2.2 [1.4, 3.4]	2.5E-04	220	157	0.4 [0.2, 0.6]	1.2E-05

Supplementary Table 6. *BECN1* and *BRCA1* expression association with subtypes among patients with high mRNA expression.

TCGA cohort

	***BECN1* expression in *BRCA1* high mRNA expression group**				***BRCA1* expression in *BECN1* high mRNA expression group**			
	**mRNA Expression**		**Odds Ratio (95% confidence intervals)**	**P Value**	**mRNA Expression**		**Odds Ratio (95% confidence intervals)**	**P Value**
	High	Low			High	Low		
**PAM50 Subtypes**								
Luminal A/B	227	121	Reference		225	174	Reference	
HER2-enriched	6	31	9.6 [3.8, 29.1]	1.1E-08	9	3	0.4 [0.07, 1.8]	0.25
Basal-like	4	78	36.3 [13.2, 139.9]	1.3E-25	5	2	0.5 [0.05, 3.2]	0.70
***TP53* Mutation**								
Wild type	192	108	Reference		188	149	Reference	
Mutant	34	104	5.4 [3.4, 8.8]	1.0E-14	38	15	0.5 [0.2, 1.0]	0.036
**Tumor Grade**								
I	17	8	Reference		11	44	Reference	
II	60	73	2.6 [1.0, 7.4]	0.049	69	102	0.4 [0.2, 0.8]	0.0059
III	22	123	11.6 [4.2, 35.3]	1.7E-07	2 4	38	0.4 [0.2, 1.0]	0.042

METABRIC cohort

	***BECN1* expression in *BRCA1* high mRNA expression group**				***BRCA1* expression in *BECN1* high mRNA expression group**			
	**mRNA Expression**		**Odds Ratio (95% confidence intervals)**	**P Value**	**mRNA Expression**		**Odds Ratio (95% confidence intervals)**	**P Value**
	High	Low			High	Low		
**PAM50 Subtypes**								
Luminal A/B	421	235	Reference		414	390	Reference	
HER2-enriched	34	102	5.4 [3.5, 8.4]	5.6E-17	38	25	0.7 [0.4, 1.2]	0.19
Basal-like	30	138	8.2 [5.3, 13.1]	5.7E-28	31	23	0.8 [0.4, 1.4]	0.48
***TP53* Mutation**								
Wild type	203	142	Reference		204	219	Reference	
Mutant	16	35	3.1 [1.6, 6.3]	2.6E-04	17	15	0.8 [0.4, 1.8]	0.71
**Tumor Grade**								
I	33	20	Reference		33	78	Reference	
II	222	149	1.1 [0.6, 2.1]	0.77	219	243	0.5 [0.3, 0.7]	9.1E-04
III	221	305	2.3 [1.2, 4.3]	0.0056	225	154	0.3 [0.2, 0.5]	4.4E-08

Supplementary Table 7. *BECN1* and *BRCA1* expression association with subtypes among patients with low mRNA expression.

TCGA cohort

	***BECN1* expression in *BRCA1* low mRNA expression group**				***BRCA1* expression in *BECN1* low mRNA expression group**			
	**mRNA Expression**		**Odds Ratio (95% confidence intervals)**	**P Value**	**mRNA Expression**		**Odds Ratio (95% confidence intervals)**	**P Value**
	High	Low			High	Low		
**PAM50 Subtypes**								
Luminal A/B	160	105	Reference		102	112	Reference	
HER2-enriched	3	27	13.6 [4.0, 71.9]	7.6E-08	34	21	0.6 [0.3, 1.1]	0.07
Basal-like	5	54	16.3 [6.3, 54.1]	5.6E-14	87	47	0.5 [0.3, 0.8]	0.0019
***TP53* Mutation**								
Wild type	132	84	Reference		90	89	Reference	
Mutant	21	95	7.1 [4.0, 12.9]	2.0E-14	120	81	0.7 [0.4, 1.0]	0.079
**Tumor Grade**								
I	42	9	Reference		7	14	Reference	
II	116	71	2.8 [1.3, 7.1]	0.0071	63	86	0.7 [0.2, 1.9]	0.49
III	41	120	13.5 [5.8, 34.3]	5.3E-13	143	101	0.4 [0.1, 1.0]	0.037

METABRIC cohort

	***BECN1* expression in *BRCA1* low mRNA expression group**				***BRCA1* expression in *BECN1* low mRNA expression group**			
	**mRNA Expression**		**Odds Ratio (95% confidence intervals)**	**P Value**	**mRNA Expression**		**Odds Ratio (95% confidence intervals)**	**P Value**
	High	Low			High	Low		
**PAM50 Subtypes**								
Luminal A/B	369	188	Reference		215	194	Reference	
HER2-enriched	25	79	6.2 [3.8, 10.5]	1.9E-15	109	68	0.7 [0.5, 1.0]	0.047
Basal-like	30	133	8.7 [5.6, 13.9]	6.9E-28	151	126	0.9 [0.7, 1.3]	0.64
***TP53* Mutation**								
Wild type	220	156	Reference		137	161	Reference	
Mutant	14	34	3.4 [1.7, 7.1]	1.7E-04	39	28	0.6 [0.3, 1.1]	0.079
**Tumor Grade**								
I	82	35	Reference		21	38	Reference	
II	248	156	1.5 [0.9, 2.4]	0.10	150	163	0.6 [0.3, 1.1]	0.089
III	147	284	4.5 [2.9, 7.3]	4.4E-12	303	275	0.5 [0.3, 0.9]	0.014
